# HIV-1 Infection Is Associated With Increased Prevalence and Abundance of *Plasmodium falciparum* Gametocyte-Specific Transcripts in Asymptomatic Adults in Western Kenya

**DOI:** 10.3389/fcimb.2020.600106

**Published:** 2021-02-05

**Authors:** Deborah M. Stiffler, Janet Oyieko, Carolyne M. Kifude, David M. Rockabrand, Shirley Luckhart, V. Ann Stewart

**Affiliations:** ^1^ Department of Preventive Medicine and Biostatistics, Division of Tropical Public Health, Uniformed Services University of the Health Sciences, Bethesda, MD, United States; ^2^ Basic Science Laboratory, US Army Medical Research Directorate–Africa/Kenya Medical Research Institute, Kisumu, Kenya; ^3^ Department of Entomology, Plant Pathology and Nematology and Department of Biological Sciences, University of Idaho, Moscow, ID, United States

**Keywords:** malaria, HIV, transmission, gametocyte, asymptomatic, co-infection, HIV-1, *Plasmodium falciparum*

## Abstract

As morbidity and mortality due to malaria continue to decline, the identification of individuals with a high likelihood of transmitting malaria is needed to further reduce the prevalence of malaria. In areas of holoendemic malaria transmission, asymptomatically infected adults may be infected with transmissible gametocytes. The impact of HIV-1 on gametocyte carriage is unknown, but co-infection may lead to an increase in gametocytemia. In this study, a panel of qPCR assays was used to quantify gametocyte stage-specific transcripts present in dried blood spots obtained from asymptomatic adults seeking voluntary HIV testing in Kombewa, Kenya. A total of 1,116 *Plasmodium*-specific *18S*-positive samples were tested and 20.5% of these individuals had detectable gametocyte-specific transcripts. Individuals also infected with HIV-1 were 1.82 times more likely to be gametocyte positive (P<0.0001) and had significantly higher gametocyte copy numbers when compared to HIV-negative individuals. Additionally, HIV-1 positivity was associated with higher gametocyte prevalence in men and increased gametocyte carriage with age. Overall, these data suggest that HIV-positive individuals may have an increased risk of transmitting malaria parasites in regions with endemic malaria transmission and therefore should be at a higher priority for treatment with gametocidal antimalarial drugs.

## Introduction

In areas of high endemicity for malaria, adults can asymptomatically maintain *Plasmodium falciparum* infection through partial immunity (Druilhe and Khusmith, 1987; Smith et al., 1999). Chronically infected asymptomatic adults and older children account for a large proportion of infected individuals, likely contributing to the sustained presence of the parasite in regions with declining malaria morbidity and mortality (Owusu-Agyei et al., 2002; Bousema et al., 2014; Sattabongkot et al., 2018). Since gametocytes are known to be intermittently present even in infections characterized by very low parasitemia, asymptomatic individuals could be a major reservoir for transmission (Schneider et al., 2007; Ouédraogo et al., 2009; Lindblade et al., 2013; Lin et al., 2014; Farid et al., 2017). Identifying individuals with an increased likelihood of transmitting parasites is a high priority for reducing the prevalence of malaria (Bousema et al., 2014).

Gametocyte prevalence within a population is known to be dependent on several factors, including age and malaria endemicity (Ouédraogo et al., 2007; Stepniewska et al., 2008; Farfour et al., 2012; Adomako-Ankomah et al., 2017), but gametocytemia has been difficult to accurately characterize because immature gametocytes sequester in the bone marrow (Rogers et al., 2000; Joice et al., 2014) and gametocytes are underreported by microscopy (Dowling and Shute, 1966; Koepfli and Yan, 2018). Molecular diagnostic techniques for gametocyte detection have been developed and are highly effective in quantifying gametocyte-specific RNA transcripts as a relative measure for gametocyte carriage (Niederwieser et al., 2000; Wampfler et al., 2013; Pett et al., 2016). The gene product *pfs25*, which is highly expressed by female stage V gametocytes, is commonly used as a marker for gametocyte carriage, as it most accurately reflects the presence of mature gametocytes that are infective to mosquitoes (Niederwieser et al., 2000; Schneider et al., 2004). Additional gametocyte-specific gene markers have been identified, including *pfs16*, which is highly expressed in the early stages of gametocyte development and remains expressed at lower levels throughout subsequent stages of development (Bruce et al., 1994; Dechering et al., 1997; Schneider et al., 2004). The gene product *pfs48/45* is expressed by intermediate-stage gametocytes, albeit at a lower level than either *pfs16* or *pfs25* (Niederwieser et al., 2000; Eksi et al., 2008; Joice et al., 2013; Aguilar et al., 2014; Chang et al., 2016; Dinko et al., 2018). Although the majority of gametocytes appear to sequester in the bone marrow during development (Rogers et al., 2000; Joice et al., 2014), low levels of transcripts for early and intermediate stages can be detected in peripheral blood (Aguilar et al., 2014).

Despite the large geographical overlap between HIV-1 and malaria prevalence, the impact of HIV-1 co-infection on malaria is poorly understood for asymptomatic adults. Much of the prior work pertaining to HIV-1 co-infection in adults with asymptomatic malaria used less sensitive methods for parasite detection, such as microscopy or rapid diagnostic tests (RDTs) (Onyenekwe et al., 2007; Iroezindu et al., 2012; Omoti et al., 2013; Njunda et al., 2016), or have been hindered by relatively small samples sizes (Berg et al., 2020). To date, co-infection with HIV-1 has been associated with a reduction in *P. falciparum*-specific antibody responses (Weiss et al., 2011; Subramaniam et al., 2015), which may impair gametocyte-specific antibodies. Some antiretroviral therapy (ART) drugs have been shown to have gametocidal properties (Hobbs et al., 2013; Hobbs et al., 2018), although these effects would not benefit individuals who are unaware of their HIV status or who are not receiving potentially gametocidal ART after a diagnosis of HIV-1 infection (Ng’ang’a et al., 2014). The only study to have examined the functional effects of immunodeficiency virus co-infection on transmissible gametocytes demonstrated that gametocytemia and associated parasite transmission to *Anopheles freeborni* were significantly increased in *Rhesus* macaques co-infected with simian immunodeficiency virus (SIV) and *Plasmodium fragile* (Trott et al., 2011). Thus, there remains a notable gap in the literature concerning the effect of HIV-1 on malaria transmission in ART-naïve, asymptomatic adults. In this study, we report on the use of a panel of gametocyte stage-specific qPCR assays to quantify gametocyte transcripts in dried blood spots (DBS) from asymptomatically infected adults seeking voluntary HIV-1 testing in Kombewa, Kenya.

## Materials and Methods

### Sample Collection in Kombewa, Kenya

Samples for this study were collected under the supervision of the Institutional Review Boards of the Uniformed Services University of the Health Sciences (USUHS, Bethesda, MD), Walter Reed Army Institute of Research (WRAIR, Silver Spring, MD), and the Kenya Medical Research Institute (KEMRI protocol SSC. No 2600) as part of a cross-sectional molecular epidemiological study investigating the relationships between HIV-1 and falciparum malaria in asymptomatic adults. Informed consent was obtained from all participants; samples were obtained as DBS from 1,762 healthy adults seeking voluntary HIV testing at Kombewa County Hospital or Manywanda Sub-County Hospital. From each patient, one to five 50 µl blood spots were blotted onto Whatman^®^ 903 Protein Saver filter paper cards (GE Healthcare Life Sciences, Chicago, IL, USA) in the outpatient laboratory. Whatman^®^ 903 Protein Saver filter paper cards have been validated as an effective method for preservation of *Plasmodium* nucleic acids when stored dry and frozen at -80°C (Jones et al., 2012), (Pritsch et al., 2012). Cards were allowed to air-dry rapidly on the air intake of a biological safety cabinet and, once dry, were placed into individual sample storage bags with desiccant packets and stored frozen at -80°C to minimize RNA degradation. At the time of sample collection, patients were also tested for HIV using the Alere Determine™ HIV 1/2 RDT (Abbott Laboratories, Chicago, IL, USA) and for malaria using Parascreen^®^ RDT (Zephyr Biomedicals, Verna, Goa, India). Subsequently, samples were tested for the presence of malaria parasite-specific transcripts using a qPCR assay that detects *18S* ribosomal RNA and DNA for human *Plasmodium* species (Kamau et al., 2011). Of the 1,133 samples found to be *18S* positive (C. Kifude, pers. comm.), 1,116 had adequate remaining DBS for gametocyte transcript analysis by qPCR. Samples collected for this study were also analyzed for the prevalence of antifolate resistance makers in the context of HIV-1 co-infection (Torrevillas et al., 2020).

### Nucleic Acid Extraction From DBS and cDNA Synthesis

DBS were cut out of filter paper cards and minced with a single use razor blade to eliminate the risk of cross-contamination. Filter paper pieces were then treated with Buffer AL, Proteinase K, and Buffer ATL (Qiagen, Hilden, Germany) to rehydrate the samples, lyse red blood cells and parasites, and degrade unwanted proteins and enzymes found in human blood. Half of the resulting lysate was used for RNA extraction using the RNeasy^®^ Mini Kit (Qiagen, Hilden, Germany), while the other half was retained for additional studies. A total of 3 µl of each RNA lysate was treated with DNase, then subjected to cDNA synthesis using the QuantiTect^®^ Reverse Transcription Kit (Qiagen, Hilden, Germany) and QuantiTect^®^ oligonucleotide primers in a 20 µl reaction following the manufacturer’s protocol. Due to limited volume and low concentration of RNA in each sample, quality assessment and quantification of RNA was not conducted prior to cDNA amplification. No-template controls (NTCs) were included during the reverse transcription step as negative controls.

### Selection of Markers and Primer and Probe Design

Novel TaqMan™ primer pairs and probes were designed against *P. falciparum* gametocyte gene sequences in PlasmoDB (https://plasmodb.org). Initially, five genes were selected: the sexual commitment transcription factor *pfAP2-G*, an early gametocyte marker *pfs16*, an intermediate gametocyte marker *pfs48/45*, a male-specific marker *pfs230p* and an abundantly expressed female-specific marker *pfs25* (**Supplementary Table 1**). Primers and probes were checked for melting temperature and lack of predicted hairpin formation using Primer Express™ 3.0 software (Applied Biosystems, Foster City, CA, USA). To ensure that primer and probe sequences were free of single nucleotide polymorphisms, sequences were evaluated with NCBI BLAST™ (https://blast.ncbi.nlm.nih.gov/). Sequences for primers (Integrated DNA Technologies, Coralville, IA, USA) and probes (Life Technologies, Carlsbad, CA, USA) are listed in **Supplementary Table 2**. Primers and probes were tested using cDNA from asynchronous *P. falciparum* NF54 gametocyte culture (data not shown) and samples from 126 *18S*-positive volunteers from the study. *pfAP2-G* was detected in one sample and *pfs230p* was not detected in any samples (data not shown). Because low parasitemias were common among our asymptomatic volunteers, we chose not to use *pfAP2-G* and *pfs230p* as markers for further analyses of our samples.

### Verification of Gametocyte Gene Expression Using Cultured *P. falciparum*

Gametocyte-specific gene expression was validated using the gametocyte-producing *P. falciparum* 3D7 strain and the gametocyte-deficient HB-2 strain (Bhasin and Trager, 1984), (BEI Resources/MR4, Manassas, Virginia, USA). Parasites were thawed from liquid nitrogen storage by dropwise addition of NaCl solutions (Moll et al., 2013) and cultured using a previously established protocol (Miao et al., 2013) adapted as follows. 3D7 and HB-2 cultures were established with 4% hematocrit. At ~5% parasitemia, predominantly ring stage parasites were synchronized using 5% D-sorbitol. This treatment was repeated after 48 h, then the volume of medium in each flask was doubled and parasites were maintained in culture for 48 h. At this point, 20 U/ml heparin (sodium salt from porcine intestinal mucosa, Sigma Aldrich, St. Louis, MO, USA) was added to the culture to inhibit merozoite invasion of RBCs (Boyle et al., 2010; Miao et al., 2013). Medium with added heparin was exchanged daily. Daily thin smears were used to monitor parasite growth and daily DBS were prepared using Whatman^®^ 903 Proteinsaver cards (GE Healthcare Life Sciences, Chicago, IL, USA) until the appearance of stage V gametocytes by microscopy or until no parasites could be observed on thin film. Each DBS was prepared from 200 µl of pelleted parasite culture that was rinsed twice in complete media to remove heparin, then resuspended at ~50% hematocrit in a final volume of 50 µl of complete medium. Whatman^®^ cards were air dried, then frozen at -80°C as described in *Sample Collection in Kombewa, Kenya*.

Daily samples of 3D7 and HB-2 cultures prepared as above were used to define transcript expression patterns of *pfs16*, *pfs48/45*, and *pfs25*. Specifically, these transcripts were quantified for 12 days, starting the day before the culture medium was doubled (**Supplementary Figure 1**). To control for variation in parasitemia among DBS samples, gDNA extracted from each sample (QIAamp DNA Mini Kit, Hilden, Germany) was used to quantify single copy *pfAQP* (Vo et al., 2007) and the ring stage marker *pfsbp1* (Farid et al., 2017) for normalization of transcript expression to genomes per µl and to monitor asexual parasitemia, respectively, in each sample. Primer and probe sequences for *pfAQP* and *pfsbp1* are listed in **Supplementary Table 2**. For both *pfAQP* and *pfsbp1*, qPCR assays were conducted using TaqMan™ Fast Universal PCR Master Mix (2X) with AmpErase™ UNG (ThermoFisher Scientific, Waltham, MA, USA) in optical plates with optical film. Each 20 µl reaction included 1 µl of template with primers and probes at 312.5 nM for *pfAQP* and 250 nM for *pfsbp1*. HB-2 cultures were discontinued after day 6 because parasites could no longer be visualized by thin smear, while 3D7 cultures were continued until day 12, at which point, stage V gametocytes were present by microscopy.

### Creation of Plasmid Standards

To quantify gene expression using qPCR, a plasmid standard was created for each gene target. The amplicon of interest was amplified from cultured *P. falciparum* 3D7 and verified by gel electrophoresis. The PCR product was then cloned into the pCR 2.1-TOPO TA vector and transformed into competent *E. coli* using the TOPO^®^ TA Cloning^®^ Kit for Subcloning with OneShot™ Top10 Chemically Competent *E. coli* (Invitrogen, Carlsbad, CA, USA). The QIAprep^®^ MiniPrep kit (Qiagen, Hilden, Germany) was used to isolate and purify plasmid DNA, which was then quantified by NanoDrop™2000C (ThermoFisher Scientific, Waltham, MA, USA). Insertion of the desired target amplicon into the plasmid was verified by Sanger sequencing conducted by Genewiz (Frederick, MD, USA). Plasmid dilutions for qPCR calibration were based on the mass of the amplicon of interest and the concentration of plasmid DNA as determined by NanoDrop™.

### Quantitative Real-Time PCR (qPCR)

All qPCR reactions were conducted using the Applied Biosystems 7500 Fast System (Applied Biosystems, Foster City, CA, USA) and TaqMan™ Multiplex Master Mix (ThermoFisher Scientific, Waltham, MA, USA). All three gametocyte markers were quantified in a single reaction in which the final concentrations of each primer and probe were 250 nM. Each plate included a series of ten-fold dilutions of a mix of the three amplicon-specific plasmids and a water (no-template) control. All samples and standards were analyzed in triplicate. Each reaction included 1 µl of cDNA, 10 µl of master mix, 250 nM of each primer and probe, and RNase-free water to 20 µl. Reactions were performed in 96 well optical plates sealed with optical film for 40–45 cycles with the following cycling parameters: 50°C for 2 min, 95°C for 10 min (one cycle) and 95°C for 15 s and annealing, extension, and detection of fluorescence at 60°C for 1 min (40 cycles).

### qPCR Assay Validation and Optimization

Ten-fold dilutions ranging from 100,000 copies per µl to 0.1 copies per µl were used to determine the limits of detection and quantification. Additional two-fold dilutions ranging from 10 copies per µl to 0.625 copies per µl were included to more accurately determine the lower limits of the assays. For all three gene markers, the lower limit of quantification was 2.5 copies per µl of template and the lower limit of detection was 1 copy per µl of template. Specificity was confirmed using cDNA from cultured *P. falciparum* 3D7 parasites (as described in *Verification of Gametocyte Gene Expression Using Cultured P. falciparum*) and uninfected human blood. To verify the absence of cross-reactivity in each triplex assay, the amplification efficiency of each target was determined in the presence and absence of primers, probes, and standards for the other two targets (data not shown) and determined to be the same as the single-plex assays (data not shown).

### Analysis of qPCR Data

To facilitate comparisons among runs, cycle threshold (Ct) values were manually adjusted to 0.2 Ct for standards and were analyzed using semi-logarithmic nonlinear regression in GraphPad Prism 6 (GraphPad, San Diego, CA, USA). Ct values for samples were interpolated using a semi-logarithmic distribution of standard values to calculate copy number per µl. Plates were excluded from analysis if NTCs were positive, if reaction efficiency was below 90% or above 105%, or if the average Ct value for any plasmid standards differed by more than two standard deviations from the aggregated Ct average for each particular dilution. Representative standard curves for all gene markers are shown in **Supplementary Figure 2**. Samples were considered positive if at least two of three replicates were positive. If only one replicate was positive, the sample cDNA was re-analyzed by qPCR and positivity was affirmed if at least three wells were positive from the two assays.

### Statistical Analyses

Data were analyzed using GraphPad Prism 6. A sample was considered to be gametocyte-positive if transcripts for at least one gametocyte-specific marker were reproducibly detected. Differences in prevalence between two groups were analyzed using Fisher’s exact test, while differences among three or more groups were analyzed using Chi-square. Differences in copy numbers were analyzed using Mann-Whitney for two groups or Dunn’s multiple comparisons tests for three or more groups.

## Results

### Prevalence of Gametocyte-Specific Transcripts in Samples From Human Volunteers

Of the 1,116 *18S*-positive samples tested for the presence of gametocyte-specific transcripts, 229 (20.5%) were positive for at least one gametocyte marker (*pfs16*, *pfs48/45*, and/or *pfs25*) (**Table 1**). This cohort of 1,116 individuals was comprised primarily of adults under the age of 30 years and included slightly more females than males (**Table 2**, **Supplementary Figure 3**). To evaluate effects of age on gametocyte transcript carriage, study participants were divided into four age brackets defined by the interquartile ranges for the cohort. For both males and females, there were significant differences in gametocyte prevalence between age brackets, as determined by Chi-squared analysis (**Table 2**), which resulted in P values of 0.047 and 0.0017 for males and females, respectively. Interestingly, the prevalence of gametocyte positive individuals was highest in the oldest age bracket for males (29.9%) but lowest in the oldest age bracket for females (12.1%, **Table 2**).

**Table 1:** Comparison of gametocyte positivity, as measured by detectable transcripts for one or more gametocyte markers, by HIV-1 status.

	*Gametocyte Positive*	*Gametocyte Negative*	*Total*
***HIV Positive***	44 (3.9%)	85 (7.6%)	129 (11.6%)
***HIV Negative***	185 (16.6%)	802 (71.9%)	987 (88.4%)
***Total***	229 (20.5%)	887 (79.5%)	1,116 (100.0%)

All 1,116 samples were positive for Plasmodium 18S by qPCR. Data are represented as numbers of samples and percentages (in parentheses) of the total cohort.

**Table 2:** Positivity for any gametocyte-specific marker (*pfs16*, *pfs48/45* or *pfs25*) by gender and age.

Age Range	Total n	Gametocyte Positive (%)	Gametocyte Negative (%)	P Value
**Male**				
18–20	129	22 (17.1)	107 (82.9)	0.047
21–24	101	21 (20.8)	80 (79.2)	
25–30	131	25 (19.1)	106 (80.9)	
31–56	147	44 (29.9)	103 (70.1)	
All Ages	508	112 (22.0)	396 (78.0)	
**Female**				
18–20	168	27 (16.1)	141 (83.9)	0.0017
21–24	155	45 (29.0)	110 (71.0)	
25–30	145	28 (19.3)	117 (80.7)	
31–56	140	17 (12.1)	123 (87.9)	
All Ages	608	117 (19.2)	491 (80.8)	

P values were calculated for each gender by comparing the number of positive and negative samples in each age bracket by Chi-square analysis.

### HIV-Positive Individuals With Asymptomatic Malaria Were More Likely to Have Gametocyte-Specific Transcripts

129 individuals (11.6% of this cohort, **Table 1**) were found to be HIV-positive by RDT. Gametocyte positivity by any marker was significantly higher in HIV-positive individuals (44/129 or 34.1% of all HIV-positive individuals in this cohort; **Table 1**) than in HIV-negative individuals (185/987 or 18.7% of all HIV-negative individuals in this cohort; **Table 1**). The relative risk of gametocyte positivity in the HIV-positive group was 1.82 times higher than that of the HIV-negative group (P<0.0001, Fisher’s exact test). In addition, the prevalences of *pfs16* and *pfs25* transcripts were significantly different between HIV-positive and HIV-negative individuals (P=0.0271 and P<0.0001, respectively, **Supplementary Tables 3 and 4**). Given that only 38 samples were positive for *pfs48/45*, prevalence by HIV-1 status was not evaluated for this marker.

### Prevalence of Individuals With Gametocyte Transcripts Was Associated With HIV-1 Status, but Age and Gender Had Little Impact on This Association

Based on the association of HIV-1 status with detectable gametocyte transcripts (**Table 1**), we sought to determine possible effects of age and gender on this association (**Supplementary Table 5**). Among all comparisons, the prevalence of individuals with detectable gametocyte transcripts differed only between HIV-negative males and females who were between 31–56 years old (P=0.0005, Fisher’s exact test), indicating that gender and age had little effect on the association of gametocyte transcript prevalence with HIV-1 status.

Because of the small sample sizes for several age brackets (**Table 2**), age groups were analyzed independently of gender for the prevalence of gametocyte transcripts by HIV-1 status and age (**Table 3**). For individuals between 31–56 years of age, there was a significant difference (P=0.0023) in gametocyte transcript prevalence between HIV-positive and HIV-negative individuals (**Table 3**). In this age bracket, the relative risk of gametocyte positivity was 2.21 times higher in HIV-positive individuals compared to HIV-negative individuals.

**Table 3:** Comparison of gametocyte-positive samples (n) by HIV-1 status and age.

Age	HIV-Positive		HIV-Negative		P Value	RR
	n	Positive (%)	n	Positive (%)		
18–20	10	2 (20.0)	287	47 (16.4)	0.67	ns
21–24	19	9 (47.4)	237	57 (24.0)	0.052	ns
25–30	48	13 (27.1)	228	40 (17.5)	0.16	ns
31–56	52	20 (38.5)	235	41 (17.4)	0.0023	2.21
Total	129	44 (34.1)	987	185 (18.7)	0.0001	1.82

Positive (%) volunteers were positive for at least one gametocyte marker. For each age range, significant differences by HIV-1 status were evaluated by Fisher’s exact test and relative risk (RR) is indicated for age brackets which were significantly different.

### HIV-Positive Individuals Had a Higher Abundance of Gametocyte-Specific Transcripts

**Figure 1:**
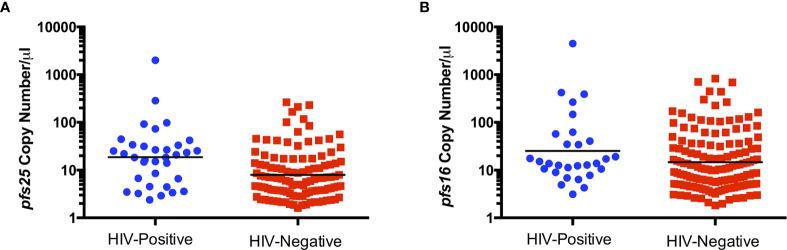
Comparison of *pfs25* **(A)** and *pfs16* **(B)** transcript copy number per µl by HIV status for combined genders and age groups. Black lines indicate geometric means. Transcript copy numbers per µl were significantly different between groups by Mann-Whitney test (*pfs25*: P=0.0006, *pfs16*: P=0.0581).

To determine whether HIV-1 status is associated with abundance of gametocyte transcripts, *pfs16*, *pfs48/45* and *pfs25* copy numbers per µl were compared between HIV-positive and HIV-negative groups. *pfs25* copy number per µl was significantly higher in HIV-positive individuals compared to HIV-negative individuals (**Figure 1A**), with a similar trend for *pfs16* (**Figure 1B**). There was no significant difference between HIV-positive and HIV-negative individuals for *pfs48/45*, perhaps due to the small number of individuals positive for these transcripts (data not shown).

### Malaria Positivity by RDT Was Associated With an Increased Risk of Gametocyte Carriage

**Figure 2:**
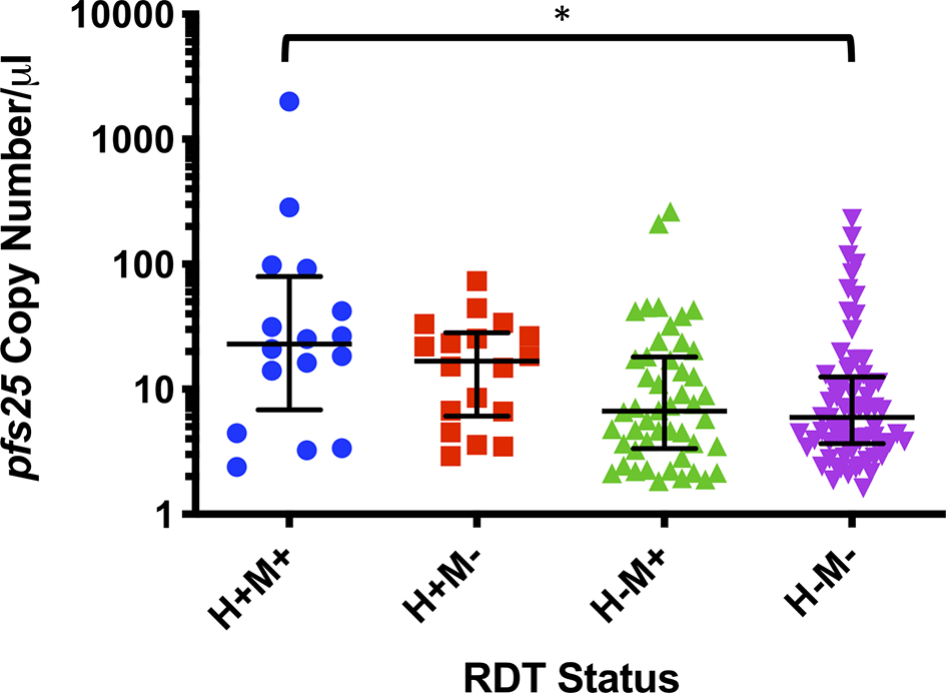
*pfs25* copy number/µl by combined HIV RDT (H) and malaria rapid diagnostic test (RDT) (M) status. Data are represented as medians and interquartile ranges and were analyzed by Dunn’s multiple comparisons test, *P ≤ 0.05.

Of the 303 samples that were malaria RDT positive, a total of 104 (34.3%) were also gametocyte positive by qPCR (**Table 4**) and this was associated with an increased risk of being gametocyte positive by any marker (RR=2.23, P<0.0001). By individual gametocyte marker, there was no difference in gametocyte copy numbers between malaria RDT positive and malaria RDT negative individuals for *pfs16* (P=0.1157, Mann-Whitney test; **Supplementary Figure 4A**) or *pfs25* (P=0.4844, Mann-Whitney test; **Supplementary Figure 4B**); *pfs48/45* was not evaluated because too few samples were positive for that marker (data not shown). However, when HIV-1 RDT positivity was included as a variable, median *pfs25* copy numbers were significantly different between individuals who were malaria RDT and HIV RDT positive and individuals who were RDT negative for both infections (**Figure 2**).

**Table 4:** Gametocyte positivity, as measured by detectable transcripts for one or more gametocyte marker, by positivity of malaria rapid diagnostic test (RDT) in samples that were *Plasmodium 18S*-positive by qPCR.

	*Gametocyte Positive*	*Gametocyte Negative*	*Total*
***RDT Positive***	104 (9.3%)	199 (17.8%)	303 (27.2%)
***RDT Negative***	125 (11.2%)	688 (61.6%)	813 (72.8%)
***Total***	229 (20.5%)	887 (79.5%)	1116 (100.0%)

Data are represented as number of samples in each group and percentage of total cohort (in parentheses). Individuals who were RDT positive were more likely to be gametocyte positive, Relative Risk=2.23, P<0.0001, Fisher’s exact test.

## Discussion

This cross-sectional cohort provided a unique opportunity to analyze prevalence of gametocyte transcript positivity in an asymptomatic and antiretroviral therapy (ART)-naïve population in a region of holoendemic malaria transmission. In this population, 20.5% of asymptomatic adults had detectable gametocyte transcripts (**Table 1**), and HIV-positive individuals were 1.82 times more likely to have detectable gametocyte transcripts (P<0.0001). HIV-positive individuals also had significantly higher gametocyte transcript copy numbers, suggesting that HIV-positive individuals may be more likely to harbor gametocytes and to maintain higher gametocytemias than HIV-negative individuals.

Gametocyte carriage has been reported to decrease with age, potentially due to the development of gametocyte-specific immunity (Adomako-Ankomah et al., 2017), but we observed this trend only in female participants (**Table 2**). Overall, male participants had a higher gametocyte prevalence, but interestingly, prevalence increased with age (**Table 2**). This effect appeared to be dependent on HIV-1 status, as separating groups by HIV-1 status resulted in almost identical gametocyte prevalence between males and females of each age group (**Supplementary Table 5**). Previous research has shown that gametocyte prevalence is reduced in individuals 35 years of age or older (Adomako-Ankomah et al., 2017), and yet, individuals between 31 and 56 years of age who were HIV-positive were at a significantly higher risk of carrying gametocyte transcripts (**Table 3**).

The specific mechanisms of increased gametocyte carriage in the context of co-infection remain to be elucidated but could be related to impaired T-cell dependent antibody responses impacting late-developing transmission blocking immunity. In particular, the finding of an increased prevalence of gametocyte transcripts in older adults suggests that HIV infection may interfere with gametocyte-specific immunity. Co-infection with *Schistosoma haematobium*, which is known to impact antibody development, has been found to be reduce some *P. falciparum* gametocyte-specific antibody levels (Ateba-Ngoa et al., 2016). HIV has been shown to modulate B cell populations and impact *P. falciparum*-specific B cells (Frosch et al., 2017), but the impact of these perturbations on gametocytes has yet to be determined. Additionally, angiogenic cytokines, including IL-1β, IL-6, and IL-8, could play a role in the release of mature gametocytes from the bone marrow (Messina et al., 2018), and these cytokines have been found to be dysregulated during HIV infection (Kedzierska and Crowe, 2001), potentially leading to the premature release of developing gametocytes. However, in the SIV and *P. fragile* model of co-infection, systemic immune activation, rather than immune dysfunction, was implicated as the cause of increased gametocytemia (Trott et al., 2011). That model, where a profound increase in gametocyte carriage in co-infected macaques was first discovered, most closely represents malaria in a naïve individual who has recently been infected with HIV. Given that our volunteers were not followed over time, longitudinal studies to capture early and later stages of co-infection are needed to understand the potential impacts of changes in host immunity on gametocyte carriage. Future studies of CD4+ T cell counts, *P. falciparum*-specific antibodies, and cytokine levels, for example, would increase our understanding of HIV-related immune dysfunction and increased gametocytemia.

This study provides insight into the utility of various molecular and clinical diagnostic techniques for measuring gametocyte carriage. RDTs, which are commonly used in the clinic, remain useful tools for the diagnosis of clinical malaria but provide little information on gametocyte carriage, because gametocyte carriage does not necessarily reflect asexual parasitemia (Koepfli et al., 2015) and can occur in the absence of high parasitemias (Farid et al., 2017). Over half of the gametocyte positive individuals in this study were malaria RDT-negative despite there being little difference in *pfs25* copy number between groups, highlighting the importance of considering gametocyte carriage and potential transmission independently of clinical presentation or RDT results. In our study, 79 samples, or 34.5% of gametocyte positive samples, were positive for only *pfs16* and/or *pfs48/45* (data not shown), markers that are expressed most highly in gametocyte stages sequestered in the bone marrow but are also expressed in mature gametocytes (Schneider et al., 2004). This finding might indicate the premature release of developing gametocytes into the circulation, but in the absence of detectable immature gametocytes by microscopy, could also be interpreted as persistent expression of these markers in circulating mature gametocytes, particularly in samples that were low-level positives for only *pfs16*. Although *pfs16* transcripts are present in committed rings (Dechering et al., 1997), we found that *pfs16* was mostly highly expressed several days after the addition of heparin to the culture media (**Supplementary Figure 1**), suggesting that early committed rings may not be the sole source of detectable *pfs16* transcripts. Interestingly, *pfs16* expression, which has been shown to peak in early gametocytes but is expressed throughout gametocyte development, was detected at higher levels than *pfs25* in mature gametocytes (**Supplementary Figure 5**), suggesting that *pfs16* could be useful for detecting mature gametocytes that are too few to be detected using *pfs25* alone.

Potential causes for the absence of gametocyte marker detection are manifold and, beyond purely technical issues, can include low levels of circulating parasites and/or parasite sequestration as well as truly negative samples. It has been particularly challenging to detect mature male gametocytes, which tend to be less abundant than female gametocytes (Annecke, 1927). Mature female gametocytes are believed to be the primary determinant of transmission to mosquitoes (Bradley et al., 2018) with the number of male gametocytes becoming more important as gametocyte density declines (Mitri et al., 2009). Although we may have underestimated gametocytemia because we did not include male-specific markers (Schneider et al., 2015), recent work has suggested, based on a high prevalence (88%) of female-biased gametocytemias among positive carriers, that analysis of *pfs25* alone is suitable for estimating gametocyte prevalence (Gruenberg et al., 2020). Several promising new targets for the detection of male gametocytes, however, may improve our understanding of the relevance of gametocyte sex ratios to transmission (Stone et al., 2017; Meerstein-Kessel et al., 2018; Wang et al., 2020). Finally, while some studies have concluded that gametocyte transcript positivity is positively correlated with mosquito infectivity (Pett et al., 2016; Adomako-Ankomah et al., 2017; Stone et al., 2017), gametocyte commitment can occur without maturity to stage V (Schneider et al., 2004) and gametocytemia is not the only predictor of mosquito infectivity (Eckhoff, 2012; Beshir et al., 2013; Nguitragool et al., 2017; Essangui et al., 2019).

The true effects of co-infection on transmission can only be determined through direct quantification of infectivity to mosquitoes. In the previously mentioned SIV/*P. fragile* model, increased oocyst counts in mosquitoes fed on blood from the co-infected animals was seen, suggesting that co-infected individuals may have enhanced infectivity to mosquitoes (Trott et al., 2011). Future studies should include standard membrane feeding assays (SMFA) to evaluate this effect in humans. Correlative studies connecting the infectivity of mosquitoes and immune cell typing in the context of malaria and HIV-1 co-infection would also be informative. In spite of the limitations of our study, this work is among the first to examine the impact of HIV-1 co-infection on falciparum gametocyte development in asymptomatic adults, and highlights the potential that asymptomatic adults co-infected with HIV-1 could serve as reservoirs of transmission in areas that are holoendemic for malaria transmission. The identification of individuals at high risk for transmitting malaria could be used to guide treatment with gametocidal drugs, further reducing the prevalence of falciparum malaria in support of ongoing efforts at elimination and eradication.

## Data Availability Statement

The original contributions presented in the study are included in the article/**Supplementary Material**. Further inquiries can be directed to the corresponding authors.

## Ethics Statement

Samples for this study were collected under the supervision of the Institutional Review Boards of the Uniformed Services University of the Health Sciences (USUHS# G18753, 4301 Jones Bridge Road, Bethesda, MD 20814 USA), Walter Reed Army Institute of Research (WRAIR #2033, 503 Robert Grant Ave., Silver Spring, MD, USA), and the Kenya Medical Research Institute (KEMRI protocol SSC# 2600, Nairobi, Kenya) as part of a cross-sectional molecular epidemiological study investigating the relationships between HIV-1 and falciparum malaria in asymptomatic adults. The patients/participants provided their written informed consent to participate in this study.

## Author Contributions

SL and VS conceived and designed the cross-sectional study. DS designed the gametocyte panel. JO and CK contributed to sample collection and analysis in Kenya. DS, CK, and DR contributed to data collection. DS performed statistical analysis and drafted the manuscript. All authors contributed to the article and approved the submitted version.

## Funding

This work was supported by the National Institutes of Health R01AI104423.

## Conflict of Interest

The authors declare that the research was conducted in the absence of any commercial or financial relationships that could be construed as a potential conflict of interest.

## Disclaimer

Material has been reviewed by the Walter Reed Army Institute of Research. There is no objection to its presentation and/or publication. The opinions or assertions contained herein are the private views of the author, and are not to be construed as official, or as reflecting true views of the Department of the Army or the Department of Defense. The investigators have adhered to the policies for protection of human subjects as prescribed in AR 70–25. The contents, views or opinions expressed in this publication or presentation are those of the author(s) and do not necessarily reflect the official policy or position of Uniformed Services University of the Health Sciences, the Department of Defense (DoD), or Departments of the Army, Navy, or Air Force. Mention of trade names, commercial products, or organizations does not imply endorsement by the U.S. Government.
