# Supplementary material for: HIV-1 Infection Is Associated With Increased Prevalence and Abundance of Plasmodium falciparum Gametocyte-Specific Transcripts in Asymptomatic Adults in Western Kenya
Source: Front Cell Infect Microbiol. 2021 Feb 5;10:600106. doi: 10.3389/fcimb.2020.600106 (PMC7892447; doi:10.3389/fcimb.2020.600106)
Supplement: Supplementary file 1 [file DataSheet_1.pdf]

Supplementary Material

1 Supplementary Figures and Tables

1.1 Supplementary Figures

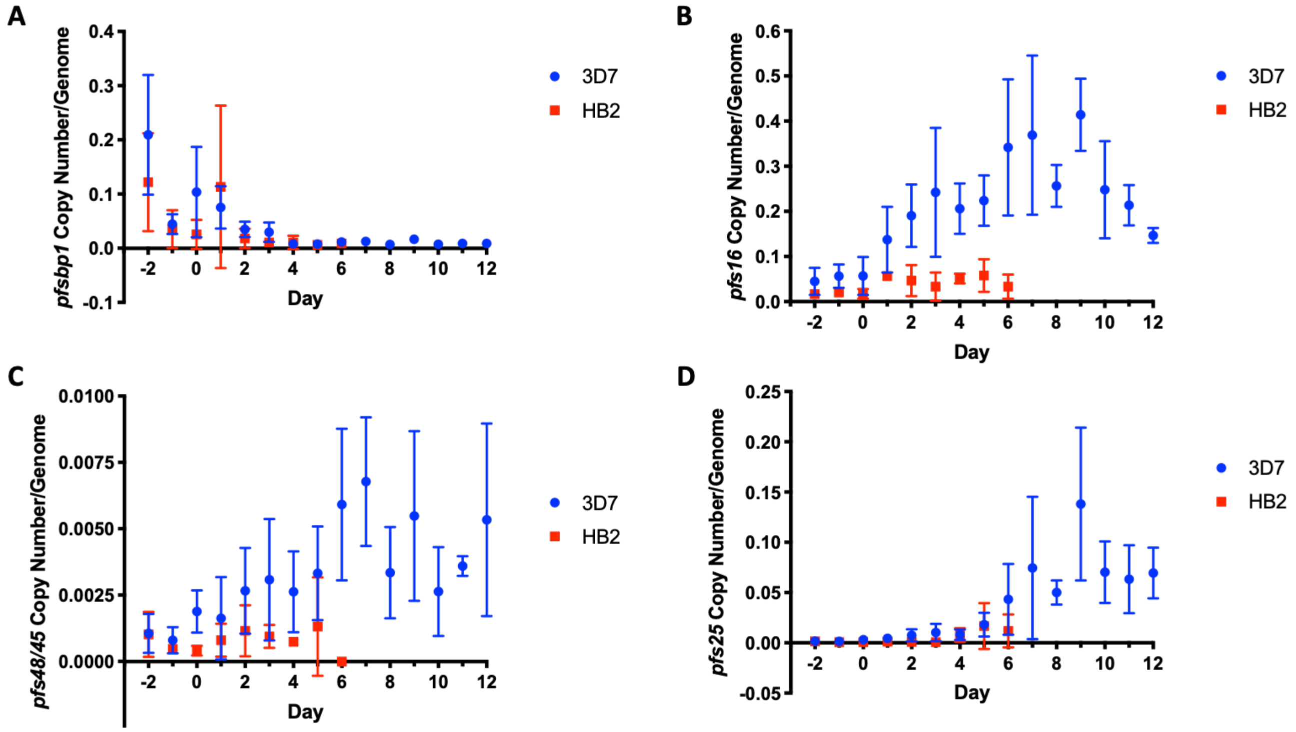

Supplementary Figure 1. Copy numbers per genome for A) *pfsbp1*, B) *pfs16*, C) *pfs48/45*, and D) *pfs25* during gametocyte development for 3D7 and HB-2 parasites. Culture medium was doubled on day -1 and heparin was added to culture medium from days 1 to 12. *pfsbp1* expression decreased after the addition of heparin to the medium on day 1 (A), while *pfs16* expression increased after the doubling of medium on day -1 (B). *pfs48/45* was expressed at higher levels during intermediate stages of gametocyte development (C), while *pfs25* expression was elevated during the late stages of gametocyte development (D). Overall, *pfs48/45* was expressed at a much lower level than the other markers.

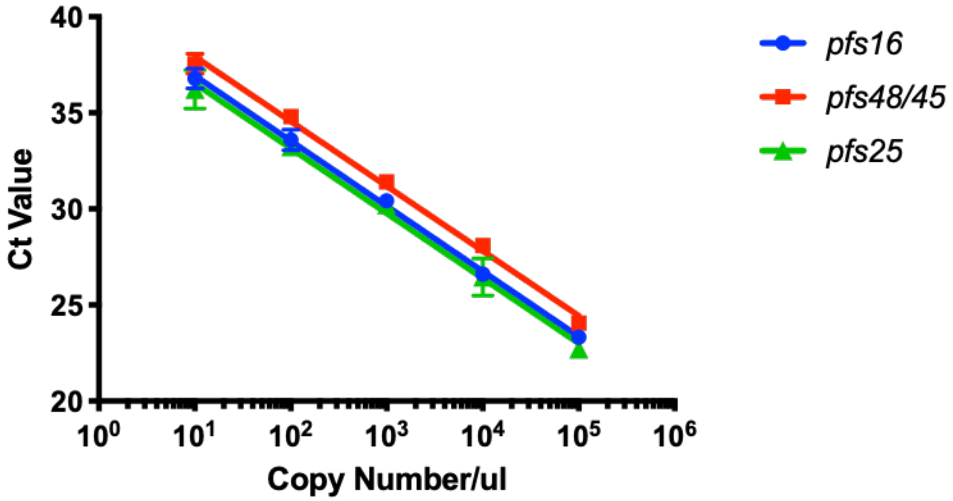

Supplementary Figure 2. Representative standard curves for *pfs16*, *pfs48/45* and *pfs25*. For *pfs16*, the slope was -3.392, $R^2 = 0.9953$ and PCR efficiency was 97.16%. For *pfs48/45*, the slope was -3.372, $R^2 = 0.9940$ and PCR efficiency was 97.95%. For *pfs25*, the slope was -3.382, $R^2 = 0.9846$ and PCR efficiency was 97.55%. Standard curves were considered acceptable if PCR efficiency was determined to be 90% to 105%.

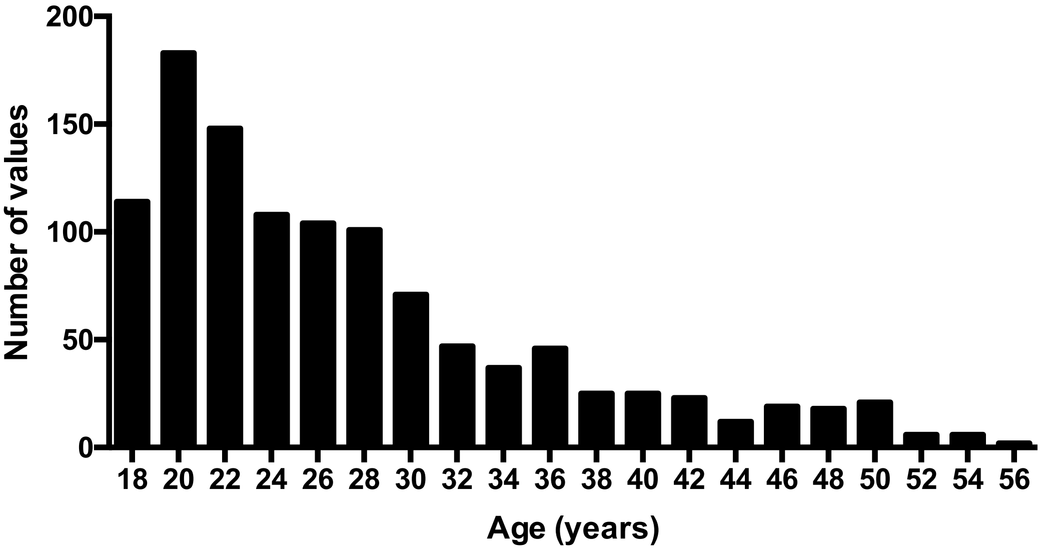

Supplementary Figure 3. Frequency distribution of age in years for the 1,116 *Plasmodium 18S*-positive samples used in this study. The study population consisted primarily of individuals 30 years of age or younger. For analysis of gametocyte prevalence by age, the study population was divided into quartiles comprised of 18-20 years, 21-24 years, 25-30 years and 31-56 years.

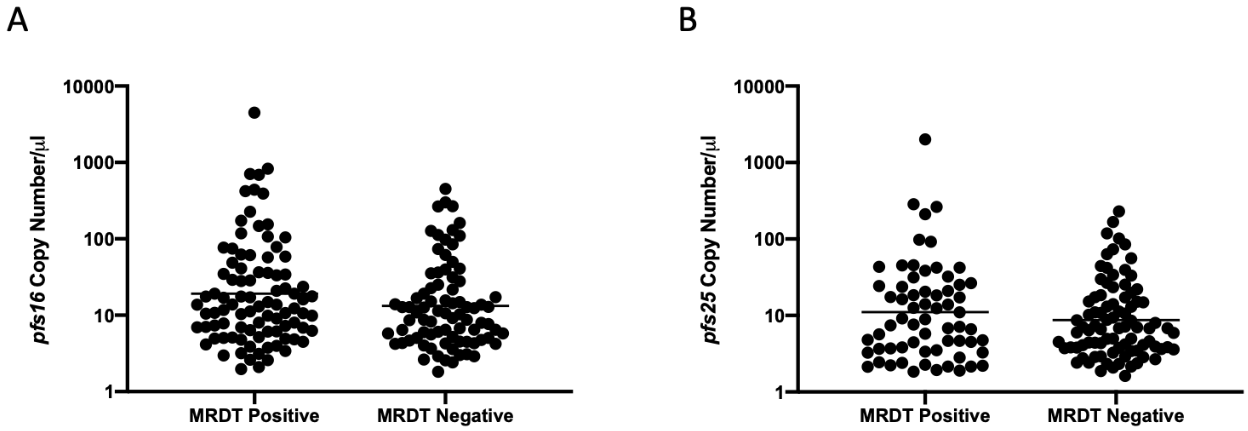

Supplementary Figure 4. Comparison of *pfs16* (A) and *pfs25* (B) transcript copy number per µl by malaria RDT (MRDT) status. Black lines indicate geometric means. Transcript copy numbers per µl were not significantly different between groups by Mann-Whitney test (*pfs16*: P=0.1157, *pfs25*: P=0.4844).

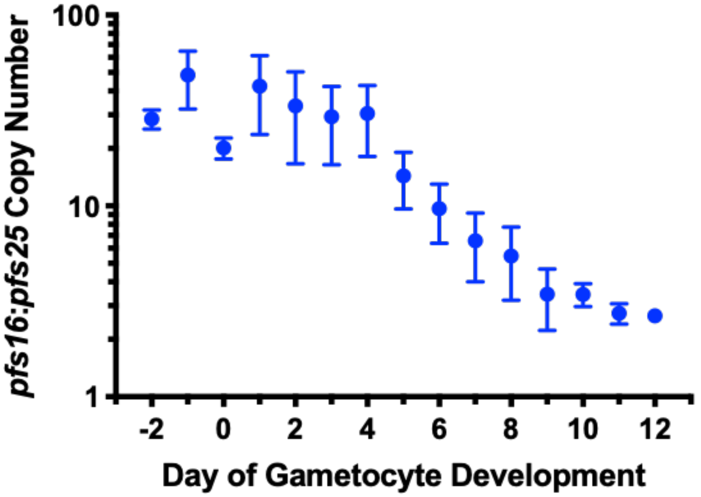

Supplementary Figure 5. Ratio of *pfs16* to *pfs25* expression over time in 3D7 parasites during gametocyte development. Although *pfs16* is considered to be an early gametocyte marker, expression levels remain higher than *pfs25* in mature gametocytes.

1.2 Supplementary Tables

Supplementary Table 1. *P. falciparum* markers, protein function, and predicted timing of mRNA expression. All markers were used to test 126 samples from *18S*-positive volunteers. *pfs16, pfs48/45* and *pfs25* were selected for characterization of the full cohort.

Marker	Protein Function	Predicted Timing of mRNA Expression
pfAP2-G	Transcriptional master regulator of gametocytogenesis (Kafsack et al., 2014)	Prior to gametocyte development, in committed parasites (Kafsack et al., 2014)
pfs16	Expressed on the membrane of the parasitophorous vacuole of gametocytes, non-essential for gametocyte development (Kongkasuriyachai et al., 2004)	Highly expressed early in gametocyte development (stages I, II); expressed at a lower level during later stages, equally expressed by male and female (Lanfrancotti et al., 2007), (López-Barragán et al., 2011), (Lasonder et al., 2016), (Bahamontes-Rosa et al., 2016)
pfs48/45	6-cysteine protein, important for male gamete fertility (van Dijk et al., 2001)	Expressed from stage II through stage V (López-Barragán et al., 2011)
pfs230p	6-cysteine protein, important for ookinete formation and transmission to mosquitoes (Marin-Mogollon et al., 2018)	Expressed by stage V male gametocytes (López-Barragán et al., 2011), (Lasonder et al., 2016)
pfs25	Ookinete surface protein (Kaslow et al., 1988)	Highly expressed by Stage V female gametocytes (López-Barragán et al., 2011), (Lasonder et al., 2016)
pfsbp1	Maurer's cleft protein, transports PfEMP-1 to the cell surface (Blisnick et al., 2000), (Maier et al., 2006)	Expressed by ring stage trophozoites (Otto et al., 2010)
pfAQP	Single copy gene encoding an aquaglyceroporin (Hansen et al., 2002)	Expressed by blood stage parasites (Hansen et al., 2002)

Supplementary Table 2. qPCR primer and probe sequences. Full-length parasite gene sequences were obtained from PlasmoDB using the GeneID listed below the target name. The 5’ end of each probe is labeled with a fluorophore (ABY, JUN, FAM, or VIC). The 3’ end of each probe is labeled with either a QSY quencher or a minor groove binder (MGB) and non-fluorescent quencher (NFQ).

Target	Forward Primer, Reverse Primer, and Probe Sequences		Amplicon Size (bp)
pfAP2-G PF3D7_1222600	Forward	5’ TTC AAC CCA AAC ATT TAA ACT TAC TCA 3’	160
	Reverse	5’ AAT CTC GAA GAT ACG ATT ATC AAC GA 3’	
	Probe	ABY 5' CGA ATG GGA AGA GAG CAT GCA ATG AAG 3’ QSY	
pfs16 PF3D7_0406200	Forward	5’ GGA TCC CCT TCA ACT TTG CA 3’	89
	Reverse	5’ CCT TGA GAT AGT CCA CCT TGA TTA GG 3’	
	Probe	JUN 5’ TTC TTC AGG TGC CTC TCT TCA TGC TGT TG 3’ QSY	
pf27/25 PF3D7_1302100	Forward	5’ AGC CCT TGG ATA AAT TTG GAA AT 3’	105
	Reverse	5’ AAA GTT GGG GAT ATT GAG TTT CAT G 3’	
	Probe	JUN 5’ AAA CAC ATG CCC CTC TCT CAC CTC GTA TT 3’ QSY	
pfs48/45 PF3D7_1346700	Forward	5’ TGT AAG CCT AGC TCT TTG AAT AGT GAA 3’	101
	Reverse	5’ TCA CGC ATA TCT GGC TTT AAA TTA TG 3’	
	Probe	VIC 5’ TAT CTG GAT TCA TAG GAT ATA AG 3’ MGBNFQ	
pfs230p PF3D7_0208900	Forward	5’ CCC AAC TAA TCG AAG GGA TGA A 3’	196
	Reverse	5’ TTA CCA AAA AAT GCT CCT AAA CGT T 3’	
	Probe	VIC 5' CAA AAC GAT CAA ACC ATC TC 3’ MGBNFQ	
pfs25 PF3D7_1031000	Forward	5’ TCT GAA ATG TGA CGA AAA GAC TGT 3’	88
	Reverse	5’ AGC GTA TGA AAC GGG ATT TCC 3’	
	Probe	FAM 5’ ATA AAC CAT GTG GAG ATT T 3’ MGBNFQ	
pfsbp1 PF3D7_0501300	Forward	5’ AAA GTA CTC CTT GTT GGC AAC GTA 3’	73
	Reverse	5’ TTA ATG AAT ACG AAG TAG AAT CTC CAG C 3’	
	Probe	FAM 5’ AAT GGC TCA AGA AGC 3’ MGBNFQ	
pfAQP PF3D7_1132800	Forward	5’ CCA TCA AGA GAT TTA GGA TCC AGA TT 3’	98
	Reverse	5’ GCT ACA AGA GGT ACC CAA AAA TAA AAA 3’	
	Probe	FAM 5’ TTG CAT ATG GAA AAG ATA CCT 3’ MGBNFQ	

Supplementary Table 3. Comparison of *pfs16* transcript prevalence by HIV-1 status in 1,116 *Plasmodium 18S*-positive samples. Data are represented as numbers of samples and percentages (in parentheses) of the total cohort. HIV-1 positivity was associated with increased *pfs16* prevalence (P=0.0271, RR=1.541, Fisher's exact test).

	***pfs16* Positive**	***pfs16* Negative**	**Total**
**HIV Positive**	29 (2.6%)	100 (9.0%)	129 (11.6%)
**HIV Negative**	144 (12.9%)	843 (75.5%)	987 (88.4%)
**Total**	173 (15.5%)	943 (84.5%)	1,116 (100.0%)

Supplementary Table 4. Comparison of *pfs25* transcript prevalence by HIV-1 status in 1,116 *Plasmodium 18S*-positive samples. Data are represented as numbers of samples and percentages (in parentheses) of the total cohort. HIV-1 positivity was associated with increased *pfs25* prevalence (P<0.0001, RR=2.243, Fisher's exact test)

	***pfs25* Positive**	***pfs25* Negative**	**Total**
**HIV Positive**	34 (3.0%)	95 (8.5%)	129 (11.6%)
**HIV Negative**	116 (10.4%)	871 (78.1%)	987 (88.4%)
**Total**	150 (13.4%)	966 (86.6%)	1,116 (100.0%)

Supplementary Table 5. Comparison of gametocyte-positive samples (n) by HIV-1 status, age and gender. Positive (%) volunteers were positive for at least one gametocyte marker. For each gender and age bracket, significant differences by HIV-1 status were evaluated by Fisher's exact test and relative risk (RR) is indicated for age brackets that were significantly different.

HIV-Positive

Age	Male		Female		P Value	RR
	n	Positive (%)	n	Positive (%)		
18-20	2	1 (50.0)	8	1 (12.5)	0.38	ns
21-24	5	2 (40.0)	14	7 (50.0)	1.00	ns
25-30	25	5 (20.0)	23	8 (34.8)	0.33	ns
31-56	40	15 (37.5)	12	5 (41.7)	1.00	ns
Total	72	23 (32.9)	57	21 (36.9)	0.58	ns

HIV-Negative

Age	Male		Female		P Value	RR
	n	Positive (%)	n	Positive (%)		
18-20	127	21 (16.5)	160	26 (16.3)	0.87	ns
21-24	96	19 (19.8)	141	38 (27.0)	0.22	ns
25-30	106	20 (18.9)	122	20 (16.4)	0.73	ns
31-56	107	29 (27.1)	128	12 (9.4)	0.0005	2.89
Total	436	89 (20.4)	551	96 (17.4)	0.22	ns

1.3 References for Supplementary Table 1

Bahamontes-Rosa, N., Gomez-Lorenzo, M.G., Lelièvre, J., Rodriguez Alejandre, A., Almela, M.J., Lozano, S., Herreros, E., and Gamo, F.J. (2016) A novel validated assay to support the discovery of new anti-malarial gametocytocidal agents. Malar J, 15:385.

Blisnick, T., Betoulle, M.E.M., Barale, J.-C., Uzureau, P., Berry, L., Desroses, S., Fujioka, H., Mattei, D., and Breton, C.B. (2000) Pfsbp1, a Maurer's cleft *Plasmodium falciparum* protein, is associated with the erythrocyte skeleton. Mol Biochem Parasitol, 111:107-121.

Hansen, M., Kun, J.F., Schultz, J.E., and Beitz, E. (2002) A single, bi-functional aquaglyceroporin in blood-stage *Plasmodium falciparum* malaria parasites. J Biol Chem, 277:4874-4882.

Kafsack, B.F., Rovira-Graells, N., Clark, T.G., Bancells, C., Crowley, V.M., Campino, S.G., Williams, A.E., Drought, L.G., Kwiatkowski, D.P., Baker, D.A., Cortés, A., and Llinás, M. (2014) A transcriptional switch underlies commitment to sexual development in malaria parasites. Nature, 507:248-252.

Kaslow, D.C., Quakyi, I.A., Syin, C., Raum, M.G., Keister, D.B., Coligan, J.E., McCutchan, T.F., and Miller, L.H. (1988) A vaccine candidate from the sexual stage of human malaria that contains EGF-like domains. Nature, 333:74.

Kongkasuriyachai, D., Fujioka, H., and Kumar, N. (2004) Functional analysis of *Plasmodium falciparum* parasitophorous vacuole membrane protein (Pfs16) during gametocytogenesis and gametogenesis by targeted gene disruption. Mol Biochem Parasitol, 133:275-285.

Lanfrancotti, A., Bertuccini, L., Silvestrini, F., and Alano, P. (2007) *Plasmodium falciparum*: mRNA co-expression and protein co-localisation of two gene products upregulated in early gametocytes. Exp Parasitol, 116:497-503.

Lasonder, E., Rijpma, S.R., van Schaijk, B., Hoeijmakers, W., Kensche, P.R., Gresnigt, M.S., Italiaander, A., Vos, M.W., Woestenenk, R., Bousema, T., Mair, G.R., Khan, S.M., Janse, C.J., Bártfai, R., and Sauerwein, R.W. (2016) Integrated transcriptomic and proteomic analyses of gametocytes: molecular insight into sex-specific processes and translational repression. Nucleic Acids Res, 44:6087-6101.

López-Barragán, M.J., Lemieux, J., Quiñones, M., Williamson, K.C., Molina-Cruz, A., Cui, K., Barillas-Mury, C., Zhao, K., and Su, X.-z. (2011) Directional gene expression and antisense transcripts in sexual and asexual stages of *Plasmodium falciparum*. BMC Genomics, 12:498.

Maier, A.G., Rug, M., O'Neill, M.T., Beeson, J.G., Marti, M., Reeder, J., and Cowman, A.F. (2006) Skeleton-binding protein 1 functions at the parasitophorous vacuole membrane to traffic PfEMP1 to the *Plasmodium falciparum*–infected erythrocyte surface. Blood, 109:1289-1297.

Marin-Mogollon, C., van de Vegte-Bolmer, M., van Gemert, G.J., van Pul, F.J.A., Ramesar, J., Othman, A.S., Kroeze, H., Miao, J., Cui, L., Williamson, K.C., Sauerwein, R.W., Janse, C.J., and

Khan, S.M. (2018) The *Plasmodium falciparum* male gametocyte protein P230p, a paralog of P230, is vital for ookinete formation and mosquito transmission. Sci Rep, 8:14902.

Otto, T.D., Wilinski, D., Assefa, S., Keane, T.M., Sarry, L.R., Böhme, U., Lemieux, J., Barrell, B., Pain, A., Berriman, M., Newbold, C., and Llinás, M. (2010) New insights into the blood-stage transcriptome of using RNA-Seq. Mol Microbiol, 76:12-24.

van Dijk, M.R., Janse, C.J., Thompson, J., Waters, A.P., Braks, J.A.M., Dodemont, H.J., Stunnenberg, H.G., van Gemert, G.-J., Sauerwein, R.W., and Eling, W. (2001) A central role for P48/45 in malaria parasite male gamete fertility. Cell, 104:153-164.
